# Pathogenesis and Therapy of Neurovascular Compression Syndromes: An Editorial

**DOI:** 10.3390/biomedicines12071486

**Published:** 2024-07-05

**Authors:** Bartosz Szmyd, Karol Wiśniewski, Dariusz J. Jaskólski

**Affiliations:** Department of Neurosurgery and Neuro-Oncology, Medical University of Lodz, Barlicki University Hospital, Kopcinskiego St. 22, 90-153 Lodz, Polanddariusz.jaskolski@umed.lodz.pl (D.J.J.)

Neurovascular compression syndromes (NVC) remains a challenging disorders resulting from the compression of cranial nerves at the transition zone. Their exact pathomechanism remains not fully elucidated. To date, many factors associated with this phenomenon have been described, encompassing the following: (1) anatomical and/or hemodynamic variability; (2) nerve alterations; (3) nucleus hyperexcitability; (4) changes in brain white and gray matter; (5) disturbances in ion channels; (6) inflammatory background; (7) altered proteome and biochemical parameters; and (8) others, such as the transaxonal short circuits theory [1,2,3,4,5,6,7,8,9,10,11,12,13,14,15,16,17,18,19]. In our previous paper, it was proposed that the most likely chain of events leading to NVC begins with vascular compression at the transition zone. This is followed by demyelination and increased nucleus excitability, which finally result in clinical symptoms [1]. This publication was a starting point for our research topic, in which we enhanced efforts leading to better insights into the NVC pathogenesis and its possible link to new therapeutic approaches. We encourage authors to submit papers related to the following aspects of pathogenesis/treatment.

Two papers focused on the anatomical variability of the superior cerebellar artery (SCA) and anterior inferior cerebral artery (AICA) in the context of neurovascular compression syndromes [20,21]. In the first of these studies, we assessed the anatomical variability of SCA specifically in the context of trigeminal neuralgia (TN). Current studies showed the following variability of this artery: duplication, single vessel origin from the posterior cerebral artery, common trunk with the posterior cerebral artery, bifurcation, and origin from the internal carotid artery [20]. Furthermore, we reviewed the AICA anatomical variability in the hemifacial spasm (HFS) context. The observed variability encompasses agenesis, duplication/triplication, fenestration, and different origin sites [21]. A single study aiming to assess vascular pattern in HFS patterns showed three different culprits, i.e., (1) a parabola-shaped loop that is vertex-oriented to the facial nerve’s REZ; (2) a large dominant AICA segment proximal to the REZ; and (3) an anchor-shaped AICA bifurcation that affects the cisternal portion of the facial nerve [22]. Nevertheless, there is no clear evidence linking selected SCA/AICA variants to a higher risk of NVC incidence [20,21]. We emphasize that neuroimaging in NVC patients should be conducted primarily for accurate differential diagnosis—such as ruling out brain tumors or vascular anomalies—rather than solely to visualize the precise site of neurovascular conflict [21]. The success rate of microvascular decompression for treating NVC largely depends on the accurate intraoperative visualization of the nerve’s REZ. Many NVC cases that are identifiable and treatable during surgery may not be visible on neuroimaging [20]. On the other hand, a radiological finding indicating potential contact between a nerve and a vessel, when not accompanied by clinical symptoms, does not justify initiating treatment for NVCs, whether pharmacological or surgical.

Further threat concerned non-surgical treatment of TN. Lee et al. pointed out that European Academy of Neurology guidelines recommend that medical management with adequate doses and regular monitoring be required before considering surgery, without specifying the optimal number of nonsurgical interventions before surgical referral. Therefore, this review covered pharmacotherapy based not only on widely used carbamazepine but also on oxcarbazepine, lamotrigine, baclofen, pimozide, tizanidine, gabapentin, pregabalin, and analgesics. In further paragraphs, the authors focused on minimally invasive procedures, i.e., nerve blocks, nerve radiofrequency and ablation, Gasserian ganglion block, Gasserian ganglion radiofrequency ablation, sphenopalatine ganglion block and radiofrequency ablation, and the administration of botulinum toxin [23].

Finally, the last paper of Carrillo-Ruiz et al. is devoted to the surgical treatment of TN. They assessed the clinical significance of the minimally invasive retrosigmoidal parasterional burr-hole approach among TN patients. Data derived from 22 patients showed significant improvement in the VAS scale ranging from 9.5 ± 0.37 before to 1.32 ± 1.28 after surgery (*p* < 0.001) and in the BNIPS scale ranging from 4.55 ± 0.25 before to 1.73 ± 0.54 after surgery (*p* < 0.001) [24].

In conclusion, we would like to express our deepest gratitude to the authors and our excellent reviewers who contributed to this research topic. We greatly encourage further efforts aiming at better understanding of NVC pathogenesis as a precondition for improving both the diagnosis and management of those entities.
